# Combining the SMAC mimetic LCL161 with Gemcitabine plus Cisplatin therapy inhibits and prevents the emergence of multidrug resistance in cholangiocarcinoma

**DOI:** 10.3389/fonc.2022.1021632

**Published:** 2022-11-30

**Authors:** Sunisa Prasopporn, Orawan Suppramote, Ben Ponvilawan, Chanette Jamyuang, Jantappapa Chanthercrob, Amphun Chaiboonchoe, Pimkanya More-Krong, Kamonchanok Kongsri, Monthira Suntiparpluacha, Rawisak Chanwat, Krittiya Korphaisarn, Seiji Okada, Somponnat Sampattavanich, Siwanon Jirawatnotai

**Affiliations:** ^1^ Siriraj Center of Research Excellence for Precision Medicine and Systems Pharmacology, Faculty of Medicine Siriraj Hospital, Mahidol University, Bangkok, Thailand; ^2^ Department of Pharmacology, Faculty of Medicine Siriraj Hospital, Mahidol University, Bangkok, Thailand; ^3^ Princess Srisavangavadhana College of Medicine, Chulabhorn Royal Academy, Bangkok, Thailand; ^4^ Hepato-Pancreato-Biliary Surgery Unit, Department of Surgical Oncology, National Cancer Institute, Bangkok, Thailand; ^5^ Division of Medical Oncology, Department of Medicine, Faculty of Medicine Siriraj Hospital, Mahidol University, Bangkok, Thailand; ^6^ Division of Hematopoiesis, Joint Research Center for Human Retrovirus Infection, Kumamoto University, Kumamoto, Japan

**Keywords:** SMAC mimetics, LCL161, Gemcitabine plus Cisplatin therapy, multidrug resistance, cholangiocarcinoma, acquired vulnerability

## Abstract

Cholangiocarcinoma (CCA) is a highly lethal gastrointestinal malignancy that has one of the worst prognoses among solid tumors. The combination of Gemcitabine + Cisplatin (GEM/CIS) remains the standard first-line treatment for advanced stage CCA. However, this drug combination yields only a modest objective response rate, and in cases that initially respond to this treatment, drug resistance commonly rapidly develops. To improve the efficiency of GEM/CIS therapy for CCA, a thorough understanding of the mechanism of GEM/CIS resistance in CCA is required. To that end – in this study, we developed several acquired GEM/CIS-resistant CCA cell lines and we screened those cell lines for acquired vulnerability. The screening process revealed that subset of CCA with GEM/CIS resistance acquired vulnerability to the small-molecule second mitochondrial-derived activator of caspases (SMAC) mimetics LCL161 and Birinapant. The observed acquired vulnerability was found to be associated with upregulation of an inhibitor of apoptosis protein 2 (cIAP2), a known target of SMAC mimetics. LCL161 or cIAP2-shRNA downregulated cIAP2 and restored the sensitivity to GEM/CIS in GEM/CIS-resistant CCA cell lines and in *in vivo* GEM/CIS-resistant xenograft models. A strong synergic effect was observed when LCL161 was added to GEM/CIS. Interestingly, this synergism was also observed in drug-naïve CCA cell lines, xenografts, and patient-derived organoids. This triplet therapy also prevented the emergence of multidrug-resistant CCA in *in vitro* and *in vivo* models. Our findings suggest that activation of cIAP2 allows CCA to escape GEM/CIS, and that suppression of cIAP2 reestablishes the apoptotic profile of CCA, thus restoring its vulnerability to GEM/CIS. The results of this study indicate that combining the SMAC mimetic LCL161 with GEM/CIS inhibits and prevents the emergence of multidrug resistance in CCA.

## Introduction

CCA is a difficult to treat cancer with high rates of recurrence and mortality. Factors that influence the unfavorable outcomes of treatment in this disease include delayed diagnosis, lack of a key molecular target, and lack of an effective and enduring pharmacologic treatment. The 5-year rate of overall survival in patients with advanced disease was reported to be as low as 2% (1). Drug resistance, both inherited and acquired, is a major problem that is responsible for the vast majority of treatment failure in CCA patients (2).

In recent years, a few oncogenic drivers have been identified as valid drug targets in CCA (i.e., mutant fibroblast growth factor receptors [FGFRs] or the isoforms 1 and 2 of isocitrate dehydrogenase [IDH1/2]); however, chemotherapy remains the key treatment modality for this disease (3). Based on the results of the Advanced Biliary Cancer (ABC)-02 trial, the combination of Gemcitabine + Cisplatin (GEM/CIS) has become the preferred first-line therapy for locally advanced or metastatic biliary tract cancer. Nonetheless, the outcome of GEM/CIS therapy is modest with an unacceptably low 28% response rate (RR) (4). The low RR is attributable to inherited or adoptive drug resistance after the treatment (5). Continued urgent study in GEM/CIS resistance in CCA is, therefore, essentially necessary, and a better understanding of the underlying mechanism of drug resistance will facilitate the development of both improved and novel therapies for CCA.

The complex mechanisms of chemoresistance in CCA facilitate the escape of cancer cells from the intended effect of anticancer agents (6, 7). Well-documented cell-intrinsic mechanisms of Gemcitabine resistance include altered drug metabolism, decreased intracellular drug concentration, and activation of prosurvival pathways, and most of these mechanisms are also observed in Cisplatin-resistant cancers (6, 8). Cisplatin resistance is also augmented by activated DNA damage repair (9). Preclinical and clinical evidence indicates that the combination of Gemcitabine and Cisplatin results in drug synergy that makes this combination therapy highly effective against several types of cancer. It has been demonstrated that Gemcitabine inhibits Akt serine/threonine kinase activity, and increases platinum-adduct retention due to decreased DNA repair when compared with Cisplatin alone (10, 11). The addition of Gemcitabine to the platinum-based therapy was shown to reverse Cisplatin resistance in several types of cancer (10, 12–14). Despite the high effectiveness of GEM/CIS therapy, the resistance to this duplet that often develops is effectuated by unknown or insufficiently well-understood mechanisms. The search for a third active drug that can synergize with GEM/CIS to prevent GEM/CIS resistance and improve treatment outcomes has started and is ongoing (4).

In the present study, we set forth to investigate the mechanism of GEM/CIS resistance, and to identify solutions that can overcome GEM/CIS resistance in CCA based on the principle of acquired vulnerability. It has been demonstrated that cancer cells under drug treatment attempt to reprofile their molecular network to survive and proliferate despite the presence of anticancer drug(s). However, this adaptation comes at a fitness cost to some collateral physical characters, which may result in an acquired vulnerability within the drug-resistant cell (15). We hypothesized that while under pressure from GEM/CIS treatment, CCA cells would rewire their molecular networks and become dependent on a new converged biological process/pathway to survive the chemotherapy. We aimed to identify the newly emerging therapeutic targets in GEM/CIS-resistant CCA cells by leveraging the acquired vulnerability of the CCA cells that grew under GEM/CIS treatment in the hope that inhibition of the acquired target would synergize with GEM/CIS and simultaneously block the escape of CCA from GEM/CIS treatment.

Our results revealed an acquired vulnerability in GEM/CIS-resistant CCA cells to second mitochondrial-derived activator of caspases (SMAC) mimetics, especially LCL161 and Birinapant. We also observed strong drug synergism between GEM/CIS and LCL161 in various CCA models, and uncovered the mechanistic basis for the observed drug synergism, which facilitates direct translation for clinical investigation of these findings.

## Materials and methods

### Cell culture and the construction of resistant cell lines

TFK1, RBE, SSP25, HuCCT1, HuCCA1, HuH28, KKU213A, KKU213B, KKU213C, KKU068, KKU131, KKU138, KKU055, KKU100, and YSCCC cell lines were obtained from the Japanese Collection of Research Bioresources (JCRB; Osaka, Japan). TFK1, RBE, SSP25, and YSCCC cells were maintained in Roswell Park Memorial Institute 1640 medium (RPMI 1640) (Gibco; Thermo Fisher Scientific, Waltham, MA, USA). HuCCA1 and KKU100 cells were maintained in Ham’s F-12 medium (Gibco; Thermo Fisher Scientific). HuCCT1, HuH28, KKU213A, KKU213B, KKU213C, KKU068, KKU131, KKU138, and KKU055 cells were maintained in Dulbecco’s Modified Eagle’s Medium (DMEM) (Gibco; Thermo Fisher Scientific). All cell lines were routinely tested for mycoplasma. For the construction of resistant cells, parental cells (KKU213C, TFK1, KKU068, and SSP25) were seeded in 12-well plates at 5 x 10^4^ cells/well in 1 milliliter (ml) of growth medium. Cells were treated with GEM/CIS using a stepwise dose-induction protocol (16).

### Drug response of CCA cell lines

Gemcitabine (cat# S1714) and Cisplatin (cat# S1166) were purchased from Selleck Chemicals (Houston, TX, USA), and LCL161 (cat# HY-15518) and Birinapant (cat# HY-16591) were from purchased from MedChemExpress (Monmouth Junction, NJ, USA). Drug response tests were performed as previously described (17).

### Drug library and high-throughput drug screening

Cells were seeded in 384-well plates at 1,000 cells/well in 40 µL of growth medium. After 24 hours, cells were treated with varied concentrations of drugs from a small Food and Drug Administration (FDA)-approved cancer drug library consisting of 62 cancer drugs (purchased from Selleck Chemicals or MedChemExpress) with 28 specific targeted molecules for 5 days. Cell numbers were determined by the Operetta^®^ High Content Screening System (PerkinElmer, Waltham, MA, USA). The images were used to calculate growth rate inhibition (GR) values using the following equation (18).

For high-throughput drug screening, cells were seeded in 384-well plates using a MultiFlo FX Multimode Dispenser (BioTek Instruments, Winooski, VT, USA) at 1,000 cells/well in 40 µl of growth medium. After 24 hours, cells were treated with the drugs in the drug library (at their respective GR_75_ values) in 20 µl of the total growth medium and using an EpMotion pipetting robot (Eppendorf, Hamburg, Germany). The cells were incubated for 5 days in a 37°C 5% CO_2_ environment. Cells were stained with 4,6-diamidino-2-phenylindole (DAPI) and imaged using an Operetta CLS™ high-content system (PerkinElmer). The nuclei number was analyzed using a Columbus Image Data Storage and Analysis System (PerkinElmer), and plotted using MATLAB 2017a software (MathWorks, Natick, MA, USA).

### Crystal violet staining

For visualization of cancer colonies, cells were washed in precooled phosphate-buffered saline (PBS), fixed with 10% neutral formaldehyde buffer for 30 minutes, and stained cells with crystal violet for 30 minutes. After staining, the cells were washed with water, air-dried for 24 hours, and imaged using a VersaDoc™ MP 4000 system (Bio-Rad Laboratories, Hercules, CA, USA).

### Drug synergistic effect

Cells were seeded in 384-well plates at 1,000 cells/well, treated with LCL161 + GEM/CIS for 5 days, stained with DAPI and imaged using an Operetta CLS™ high-content system. The nuclei number was analyzed using a Columbus Image Data Storage and Analysis System (PerkinElmer). The combination index (CI) was calculated using CompuSyn software version 1.0 (ComboSyn, Inc., Paramus, NJ, USA) and Chou-Talalay’s equation (19). The CI < 1, CI =1, and CI > 1 indicate synergism, additive effect, and antagonism, respectively.


$$\text{CI}=\frac{{\left(\text{D}\right)}_{1}}{{\left({\text{D}}_{\text{x}}\right)}_{1}}\;+\;\frac{{\left(\text{D}\right)}_{2}}{{\left({\text{D}}_{\text{x}}\right)}_{2}}=\frac{{\left(\text{D}\right)}_{1,2}\left[\frac{\text{P}}{\text{P}+\text{Q}}\right]}{{\left({\text{D}}_{\text{m}}\right)}_{1}{\left[\frac{{\text{f}}_{\text{a}}}{1-{\text{f}}_{\text{a}}}\right]}^{1/{\text{m}}_{1}}}\;+\;\frac{{\left(\text{D}\right)}_{1,2}\left[\frac{\text{Q}}{\text{P}+\text{Q}}\right]}{{\left({\text{D}}_{\text{m}}\right)}_{2}{\left[\frac{{\text{f}}_{\text{a}}}{1-{\text{f}}_{\text{a}}}\right]}^{1/{\text{m}}_{2}}}$$

### Western blotting and antibodies

Western blotting was performed as previously described (17). The following specific antibodies were used: XIAP (1:1000; cat. no. 2042; Cell Signaling Technology, Danvers, MA, USA), cIAP2 (1:1000; cat. no. 3130; Cell Signaling Technology), p-NF-kB (1:1000; cat. no. 3033; Cell Signaling Technology), NF-kB (1:1000; cat. no. 8242; Cell Signaling Technology), cIAP1 (1:1000; cat. no. sc-271419; Santa Cruz Biotechnology, Dallas, TX, USA), and β-actin (1:1000; cat. no. sc-47778; Santa Cruz Biotechnology).

### siRNA knockdown

Small interfering RNAs (siRNAs) were transfected using RNAiMAX Reagent (Thermo Fisher Scientific) according to the manufacturer’s protocol. The target sequences for cIAP2 genes were (a) 5’-AATTGGGAACCGAAGGATAAT-3’, (b) 5’-CAAGAACATGATGTTATTAAA-3’, and (c) 5’-CACTACAAACACAATATTCAA-3’ (Qiagen, Hilden, Germany). AllStars Negative Control siRNA was used as nontargeting control (cat. no. 1027281; Qiagen, Hilden, Germany). At 48 hours after sicIAP2 transfection, the resistant cells were treated with GEM/CIS for 72 hours.

### Apoptosis detection

To evaluate caspase 3 activity, cells were seeded in 12-well plates at 1.5-1.7 x 10^5^ cells/well in 1 ml of growth medium before treated with LCL161. After 72 hours, caspase3^+^ cells were detected using an FITC Active Caspase 3 Apoptosis Kit (BD Biosciences, San Jose, CA, USA) and a CytoFLEX Flow Cytometer (Beckman Coulter, Brea, CA, USA). The data analysis was performed using FlowJo™ software version 10.7.1 (FlowJo, LLC, Ashland, OR, USA). For propidium iodide (PI) (Abcam, Cambridge, UK, ab14085) permeabilization assay, cells were seeded in 12 well-plate approximately 1.5-1.7 x 10^5^ cells/well in 1 ml of growth medium. After 36 hours of LCL161 treatment, cells were harvested and washed in pre-cooled PBS 2 ml, centrifuging at 300 x g, 4°C for 5 minutes and then decanting, 2.5 μL of PI (50 μg/mL) were added and incubated in the dark for 5 minutes before proceeding to fluorescence detection by flow cytometry using CytoFLEX Flow Cytometer. Data were analyzed using CytExpert software version 2.1.0.92 (Beckman Coulter Life Sciences, Indianapolis, IN, USA).

### ROS measurement

ROS measurement was performed in 12-well plates. Cells were seeded at a density of 5×10^4^ cells/well. At the detection time, cells were washed twice with PBS, and carboxy-DCFDA dye in serum-free medium was added at a final concentration of 10 µM. Plates were incubated at 37°C for 30 min, before removal of the dye. Cells were then washed twice with PBS, and immediately detection by flow cytometry using CytoFLEX.

### Focus formation by immunofluorescence

The cells were fixed with 4% paraformaldehyde in PBS, washed and permeabilized by 0.1% Triton X-100 in PBS, and non-specific binding was blocked by the Odyssey^®^ blocking buffer. Anti γH2AX, Ser139 (Cell Signaling Technology) was used to detect γH2AX^+^ cells. Alexa fluor 647 donkey anti-mouse IgG (Thermo Fisher Scientific) was used as secondary antibody. The nuclei were counter-stained using DAPI. Images were acquired using the Operetta CLS™ high-content system (PerkinElmer). At least 300 cells were counted. Positive foci formation of γH2AX, cells were determined by more than 3 foci per cell.

### Reverse phase protein array (RPPA)

Preparation of cell lysates was performed following the protocol by RPPA Core Facility at MD Anderson Cancer Center (Houston, TX, USA). Positive control lysate was prepared from mixed cell lysates. Dilution buffer was used as negative control. Serially diluted lysates were spotted onto sixteen pad nitrocellulose‐coated slides (Grace Bio-Labs, Bend, OR, USA) by Arrayjet (Edinburgh, UK). Each pad was probed with a validated primary antibody (20). The antibodies used in this work were selected from the list from the RPPA Core Facility at MD Anderson Cancer Center. The relative fluorescence intensities of the antibody signals were detected by InnoScan 710-IR (Innopsys Inc., Chicago, IL, USA) using the Mapix software (Innopsys Inc). Signal intensities were normalized spot-by-spot division of antibody signal intensity by a housekeeping protein using RPPanalyzer (21). To analyze the results, binary-logarithm transformed median fluorescence intensities with background correction were performed a data normalization by spot-by-spot division of housekeeping protein intensities [x]. Subsequently, to investigate essential antibodies for LCL161 responses in the late time phase, partial least square discriminant analysis or PLS-DA (plsda function of mixOmics package version 6.20.0) was performed by utilizing all antibodies’ area under the curve (AUC) values calculated by using trapezoidal integration (trapz function of pracma library version 1.9.9). Then, the antibodies with VIP (variable importance in projection) scores which is close to or more than one was identified as meaningful variables for LCL161 responses among these five cell lines.

### Drug response in primary cell line, and patient-derived organoids

The study protocols were approved by the Institutional Review Board for Human Research of the Faculty of Medicine Siriraj Hospital, Mahidol University, and The National Cancer Institute, Thailand (SI494/2019 and NCI006/2020). Primary cell SiK03 was isolated from a fresh specimen that was minced and incubated with collagenase for 1 hour at 37°C, with 5% CO2. The cells were cultured in DMEM. Organoid preparation was performed according to a previous published protocol (17). Briefly, the CCA tissues Si_003 and NCI_001 were washed with cold PBS containing 10% Antibiotic/Antimycotic (Thermo Fisher Scientific) 5-10 times. The tissues were then minced using surgical blades and washed using washing media (Advanced DMEM/F12 containing 1X Glutamax™, 1X HEPES, and 1X Antibiotic/Antimycotic) (all purchased from Thermo Fisher Scientific). The tissue paste was collected *via* centrifugation at 400 x g at 4°C for 5 minutes, and further digested with 2 mg/ml collagenase D at 37°C with 5% CO_2_ for 30 minutes. Undigested tissues were filtered out using 100 µm cell strainers (Thermo Fisher Scientific). The cells were collected by centrifugation and embedded in 60% Matrigel^®^ (Thermo Fisher Scientific). The gel was solidified at 37°C with 5% CO_2_ for 30 minutes, and then organoid culture media (22) was added to each well. The cells were then incubated at 37°C with 5% CO_2_ until the organoids formed.

For the drug testing experiment, the organoids were collected and dissociated into smaller sizes *via* incubation with TrypLE™ Express Enzyme (12604021; Gibco; Thermo Fisher Scientific) at 37°C with 5% CO_2_ for 5 minutes. After incubation, the enzyme was diluted with wash media, and the cells were collected by centrifugation and counted. The cells were seeded in 384-well plates at 1,000 cells/well, suspended in organoid culture media, and incubated for 72 hours. The following final concentrations of GEM/CIS at 10/100, 1/10, 0.1/1, 0.01/0.1, 0.001/0.01, 0.0001/0.001, or 0.00001/0.0001 µM; LCL161 at 160, 16, 1.6, 0.16, 0.016, 0.0016, or 0.00016 µM; or, LCL161 + GEM/CIS at a 1:1 ratio was added and incubated under for 5 days. Cell viability was measured using an ATPlite™ Luminescence Assay System (PerkinElmer) according to the manufacturer’s instructions.

### *In vivo* studies

The protocols for all *in vivo* experiments in this study were approved by the Faculty of Medicine Siriraj Hospital, Mahidol University – Institute Animal Care and Use Committee. The BALB/C*^Rag2-/-,Jak3-/-^* mice used in this study were obtained from Kumamoto University. The mice were maintained in a 12 hour-12 hour light-dark cycle, 25°C environment, and mice were given free access to standard mouse pellets and water. CCA cell lines were subcutaneously injected into the anesthetized mice. The animals were anesthetized by intraperitoneal (I.P.) injection of 100 mg/kg ketamine: 10 mg/kg xylazine. GEM/CIS (20 mg/kg/2.5 mg/kg dose) (23) treatment was administered *via* intraperitoneal injection twice a week, and LCL161 (10 mg/kg dose) (24) was administered *via* I.P. injection every two days. Tumor length, width, and body weight were measured every other day. Tumor volume was calculated using the equation: V = ½ (Length × Width^2^). Toxicity was evaluated by mean percentage of weight loss in each group. At the experimental endpoint (when tumor size reaches 2 cm in diameter, or the animal loss 20% of the body weight), the mouse will be euthanized by I.P. injection of 300 mg/kg ketamine: 30 mg/kg xylazine.

### Statistical analysis

Data are shown as mean ± standard deviation (SD) from at least 3 experiments. All statistical analyses and paired *t*-tests were performed using GraphPad Prism version 7.0 (San Diego, CA, USA). A *p*-value of less than 0.05 was considered to be statistically significant.

## Results

### Acquired vulnerability screening in GEM/CIS-resistant CCA cell lines revealed the hypersensitivity of drug resistant CCA to small molecule SMAC mimetics

**Figure 1:**
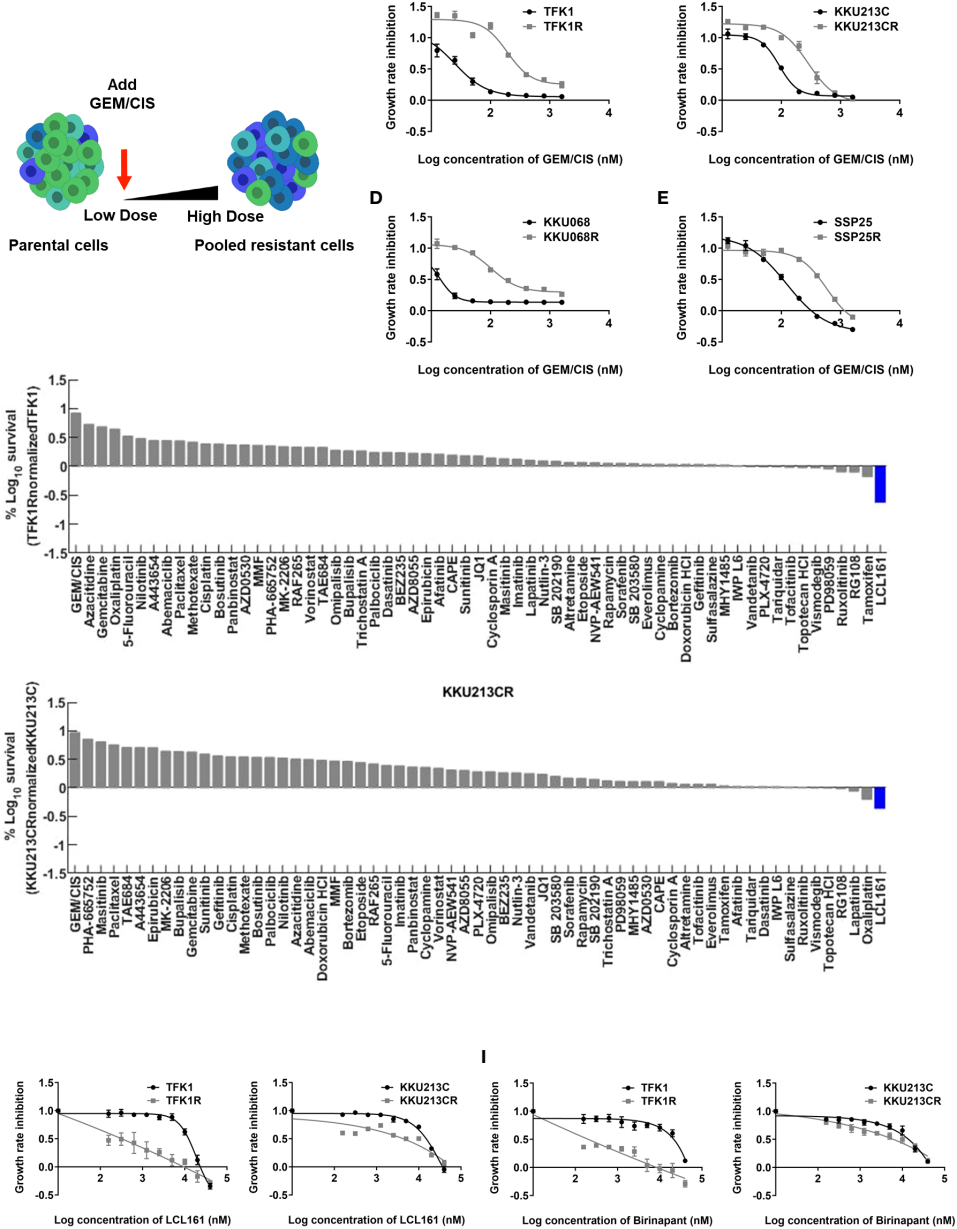
Acquired vulnerability of GEM/CIS-resistant cells to LCL161. **(A)** Parental CCA cells were cultured under increasing doses of GEM/CIS until becoming GEM/CIS-resistant cells using a step-wise dosing protocol. **(B–E)** Dose-response curves for TFK1 and TFK1R cells, KKU213C and KKU213CR cells, KKU068 and KKU068R cells, and SSP25 and SSP25R cells, respectively. The cell lines were treated with different concentrations of GEM/CIS for 5 days to generate the dose-response curves. The error bars represent the SD of four cultures. **(F)** TFK1R cells and **(G)** KKU213CR cells were screened with a variety of drugs at GR_75_ for 5 days, and the result shows the percent survival of the resistant CCA cell lines normalized to their parental drug-naïve counterparts. LCL161, which was shown to be the most effective drug, is highlighted in blue. **(H, I)** Dose-response curves for TFK1, TFK1R, KKU213C, and KKU213CR cells under LCL161 or Birinapant treatment. The error bars represent the SD of four cultures.

To develop GEM/CIS-resistant CCA models, we grew several CCA cell lines (i.e., TFK1, KKU213C, KKU068, and SSP25) under increasing doses of GEM/CIS for 4 months (**Figure 1A**). The developed GEM/CIS-resistant cell lines (TFK1R, KKU213CR, KKU068R, and SSP25R) were resistant to GEM/CIS and proliferated under higher doses of GEM/CIS compared to the parental cell lines (**Figure 1B–E**, and **Supplementary Table 1A**). GR50s of GEM and CIS in parental CCA cell lines are provided in **Supplementary Table 1B**. We performed acquired vulnerability screening on the resistant cell lines in parallel with the parental drug-sensitive cell lines. To facilitate a clinical translation, we selected 62 anticancer drugs that are in clinical trial or on the FDA-approved drug list for our drug screenings (17) (**Supplementary Figure 1A**). An acquired vulnerability hit was defined as a drug being 2 times more effective at inhibiting GEM/CIS-resistant cells compared to its GEM/CIS-sensitive counterpart.

From our screenings, we found LCL161 (25), which is an SMAC mimetic drug, to be a positive hit in 2 pairs of cells in our screening model (TFK1/TFK1R and KKU-213C/KKU213CR) (**Figure 1F, G** **)**.

We confirmed acquired vulnerability to LCL161 in TFK1R and KKU213CR cells *via* colony survival assay (**Supplementary Figure 1B**), and generated dose-response curves in those cell lines (**Figure 1H**). TFK1R and KKU213CR also acquired sensitivity to another SMAC mimetic drug – Birinapant (26) (**Figure 1I**, **Supplementary Table 2**), which suggests that the newly acquired sensitivity is to the SMAC mimetic drug class and not specific to only LCL161. Of note, the other two evaluated cell line pairs (KKU-068/KKU-068R and SSP25/SSP25R) did not yield acquired vulnerability to LCL161, Birinapant (**Supplementary Figures 1C–E** and **Supplementary Table 2**), or to any drug in our small drug library (data not shown), implying that other unknown mechanisms of drug resistance may play roles in these cells.

### LCL161 synergizes with GEM/CIS in inhibiting both GEM/CIS-resistant and parental CCA cell lines

**Figure 2:**
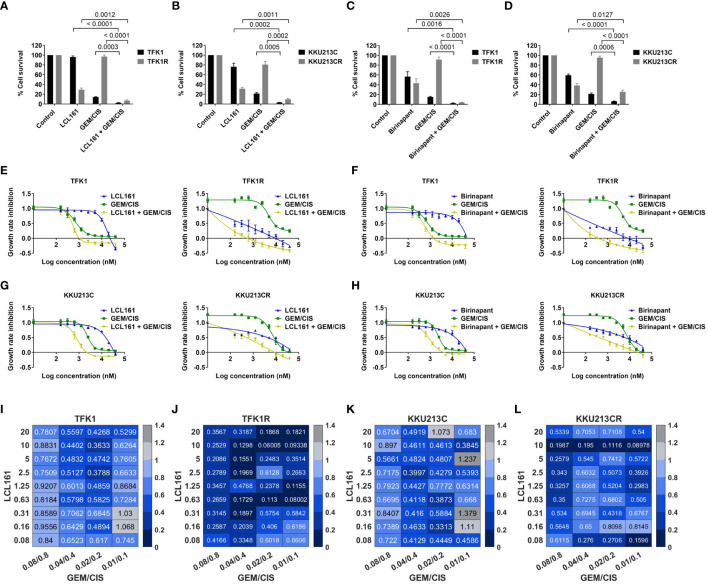
LCL161 synergistically enhances the drug efficacy of GEM/CIS. **(A, B)** Triplet LCL161 + GEM/CIS therapy effectively inhibits multidrug-resistant cells. Percent cell survival of TFK1, TFK1R, KKU213C, and KKU213CR cells treated with LCL161 at 2.5 µM, or GEM/CIS at 0.01/0.1 µM, or LCL161 + GEM/CIS, and of **(C, D)** TFK1, TFK1R, KKU213C, and KKU213CR cells treated with Birinapant at 2.5 µM, GEM/CIS at 0.01/0.1 µM, or Birinapant + GEM/CIS for 5 days. The bars represent the averages of 4 replicates ± SD. Analysis for statistical significance was performed using Student’s *t*-test, and the *p*-values are shown above the bars in all 4 images. **(E)** Dose-response curves for TFK1 and TFK1R cells treated with LCL161, GEM/CIS, or LCL161 + GEM/CIS. **(F)** Dose-response curves for TFK1 and TFK1R cells treated with Birinapant, GEM/CIS, or Birinapant + GEM/CIS. **(G)** Dose-response curves for KKU213C and KKU213CR cells treated with LCL161, GEM/CIS, or LCL161 + GEM/CIS. **(H)** Dose-response curves for KKU213C and KKU213CR cells treated with Birinapant, GEM/CIS, or Birinapant + GEM/CIS. **(I–L)** The combination indexes (CIs) of LCL161 + GEM/CIS in TFK1, TFK1R, KKU213C, and KKU213CR cells. The colors indicate the proportion of respondents. A CI < 1, CI = 1, and CI > 1 indicate synergism, additive effect, and antagonism, respectively.

Since the screening results showed the GEM/CIS-resistant CCA cells to be hypersensitive to SMAC memetics, we investigated whether the addition of SMAC mimetics to GEM/CIS produces a synergistic effect. As shown in **Figures 2A, B**, the parental cells (TKF1 and KKU-213C) were not sensitive to LCL161, and the resistant CCA cells (TFK1R and KKU-213CR) were resistant to GEM/CIS. Addition of LCL161 to the GEM/CIS duplet effectively inhibited both parental GEM/CIS-sensitive cells and GEM/CIS-resistant cells. Similar results were observed when Birinapant was added to the GEM/CIS duplet (**Figures 2C, D** **)**. These results suggest that when under pressure from GEM/CIS treatment, these cell lines reprofile themselves to survive the killing effect exerted by GEM/CIS and become reliant on the small molecule SMAC mimetic targets – inhibitor of apoptosis (IAP) proteins.

To examine whether the interaction between SMAC mimetics and GEM/CIS reflects synergism, we constructed dose-response curves for LCL161 + GEM/CIS or Birinapant + GEM/CIS. We found that the addition of LCL161 or Birinapant to GEM/CIS therapy significantly reduced the GR_50_ of GEM/CIS in the resistant cells, as well as in the parental cells (**Figures 2E–H**). The combination indexes (CIs) revealed that almost all of the evaluated doses of the LCL161 + GEM/CIS combination generated synergistic effect (all CI<1) (**Figures 2I–L**). The observed synergism was found to be stronger in the GEM/CIS-resistant cells (TFK1R and KKU213CR) (**Figures 2J, L** **)** than in the parental cells (**Figures 2I, K** **)**.

### Upregulation of cIAP2 in GEM/CIS-resistant CCA facilitates acquired vulnerability to SMAC mimetics

**Figure 3:**
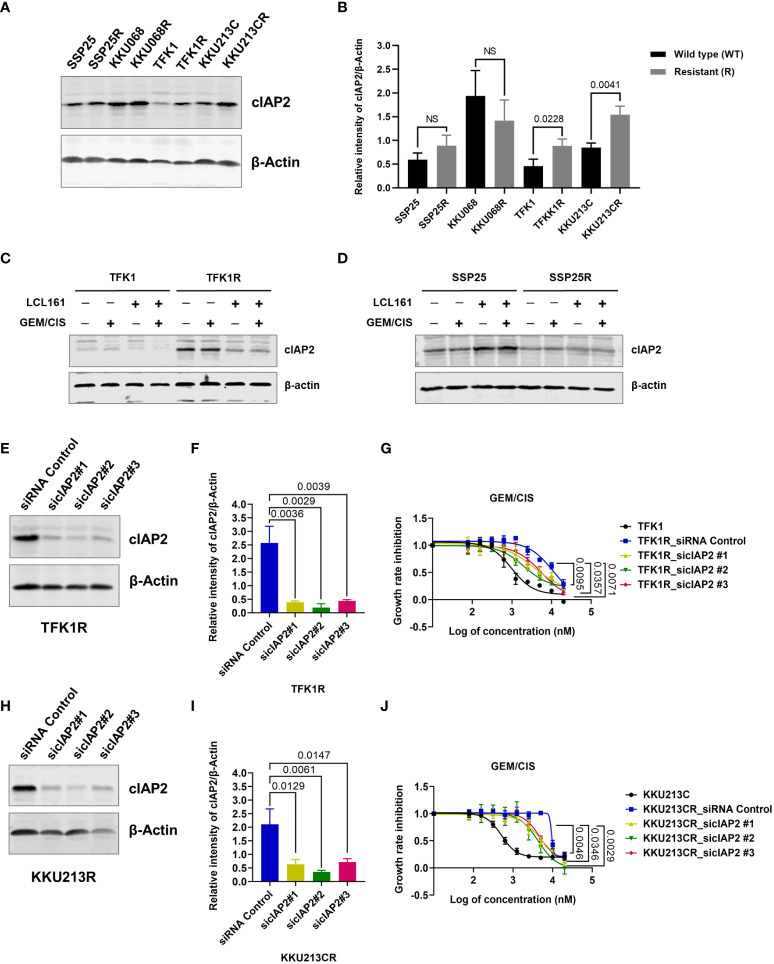
cIAP2 overexpression mediates GEM/CIS resistance in CCA. **(A)** Western blot analysis of cIAP2 in parental cells (TFK1, KKU213C, KKU068, and SSP25 cells), compared to their resistant counterparts (TFK1R, KKU213CR, KKU068R, and SSP25R cells). β-actin was used as a protein loading control. A representative result is shown. **(B)** The bar graphs show the average intensities of 3 replicates ± SD of cIAP2 expression in the indicated cell lines from **(A)** *P*-values are shown above the bars. **(C, D)** Western blot analysis of cIAP2 in TFK1 *vs.* TFK1R, and SSP25 *vs.* SSP25R cells under 72 hours of treatment with LCL161, GEM/CIS, or LCL161 + GEM/CIS, as indicated. **(E)** Western blot analysis of the cIAP2 protein in TFK1R cells transfected with the indicated siRNAs. β-actin was used as a protein loading control. A representative result is shown. **(F)** The bar graph shows the average intensities of 3 replicates ± SD of the results in **(E)** *P*-values are shown above the bars. **(G)** GEM/CIS dose-response curves for siRNA-mediated cIAP2-depleted TFK1R cell lines. The curves represent the averages of 3 replicates ± SD. *P*-values are shown at the lower right quadrant of the image. **(H)** Western blot analysis of the cIAP2 protein in KKU213CR cells transfected with the indicated siRNAs. β-actin was used as a protein loading control. A representative result is shown. **(I)** The bar graph shows the average intensities of 3 replicates ± SD of the results in **(H)** *P*-values are shown above the bars. **(J)** GEM/CIS dose-response curves for siRNA-mediated cIAP2-depleted KKU213CR cells. The curves represent the averages of 3 replicates ± SD. *P*-values are shown at the lower right quadrant of the image. NS, not statistically significant.

Since GEM/CIS-resistant CCA cell lines were found to have acquired vulnerability to SMAC mimetics, we examined the expressions of the SMAC mimetic target proteins XIAP, cIAP1, and cIAP2 in the resistant CCA cell lines *via* immunoblotting. We found that the resistant cell lines TFK1R and KKU213CR showed upregulation of cIAP2 when compared to their parental counterparts (**Figures 3A, B** **)**. The upregulation of cIAP2 in TFK1R and KKU213CR cells was found to be correlated with the upregulation and activation of NF-kB, which is a major IAP target protein (27, 28) (**Supplementary Figure 2A**). In contrast, the expression of cIAP2 and NF-kB in the GEM/CIS-resistant cell lines that did not develop acquire vulnerability to LCL161 (SSP25R and KKU068R) remained unchanged (**Figures 3A, B**, **Supplementary Figure 2A**). Using RPPA, we found that upregulation of cIAP2 in the LCL161-sensitive cell lines also associated with the activations of pathways implicated in cell survival under DNA damage-induced cellular stress, such as apoptosis, DNA damage respond (DDR), PI3K/AKT, and mTOR/TSC (**Supplementary Figure 2B**).

LCL161 treatment alone or in combination with GEM/CIS significantly suppressed cIAP2 expression in the resistant TFK1R cells (**Figure 3C**). In contrast, LCL161 had no effect in the resistant cell line SSP25R, which was not sensitive to LCL161 (**Figure 3D**).

**Figure 4:**
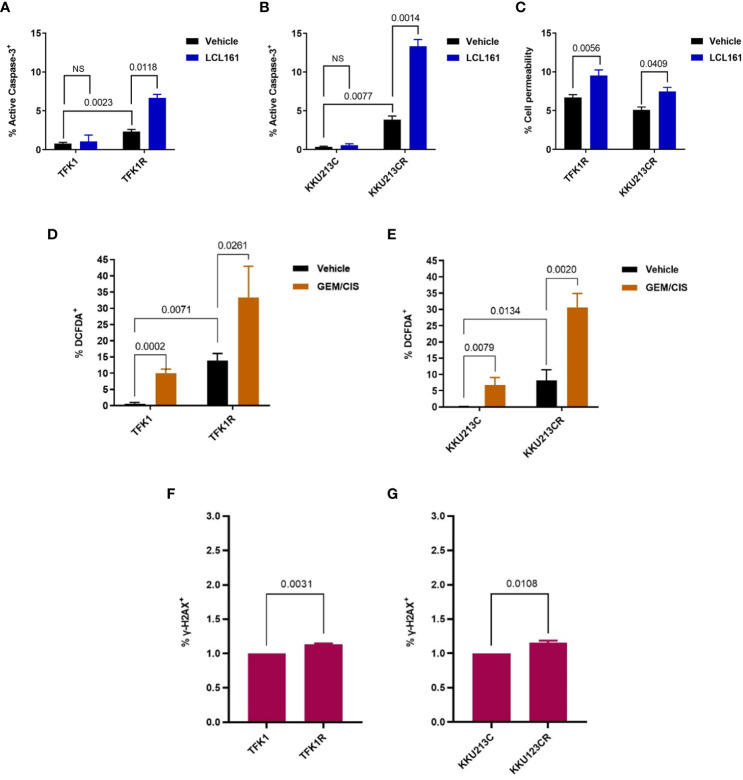
LCL161-induced apoptosis in TFK1R and KKU213CR cells. **(A, B)** Percentage of activated caspase 3^+^ in TFK1, TFK1R, KKU213C, and KKU213CR cells treated with LCL161 or vehicle at 72 hours. **(C)** Percentage of PI cell permeability in KKU213CR and TFK1R cells treated with LCL161 at 36 hours. **(D)** Percentage of DCFDA^+^ TFK1 and TFK1R cells, with GEM/CIS or vehicle at 72 hours. **(E)** Percentage of DCFDA^+^ KKU213C and KKU213CR cells, with GEM/CIS or vehicle at 72 hours. **(F, G)** Percentage of γ-H2AX^+^ TFK1, TFK1R, and KKU213C, KKU213CR cells. Bars show the mean of 3 replicates ± SD. *P*-values are shown. NS, not statistically significant.

To validate whether the expression of cIAP2 is the key mediator that facilitates the survival of GEM/CIS-resistant cells, we depleted cIAP2 expression by using cIAP2-specific siRNAs. We found that 3 independent sequences of cIAP2 siRNA, which knocked down cIAP2 expression by more than 80% (**Figures 3E, F** **)**, reproducibly suppressed TFK1R cell survival under GEM/CIS treatment (**Figure 3G**) and lowered the GEM/CIS GR_50_ values when compared to the control non-target siRNA (**Supplementary Table 3**). Similar results were confirmed in KKU213CR cells (**Figures 3H–J**, and **Supplementary Table 4**). Of note, although the reversals of GEM/CIS sensitivity were significant and reproducible, they were not complete reversals (**Figures 3G, J**: compared to parental cells in black lines), which suggests that there might be other mechanisms that influence GEM/CIS resistance. From these results, we concluded that cIAP2 is a key protein in CCA that, at least partially, facilitates CCA survival under GEM/CIS treatment. Suppression of cIAP2 *via* siRNA or SMAC-dependent degradation was enough to resensitize the CCA cells to GEM/CIS treatment. Consistent with the role of cIAP2 as an anti-apoptotic protein, we found that LCL161 promoted substantial apoptosis in the cells as demonstrated by the increase in both the number of cells with activated caspase 3 and the number of propidium iodide^+^ cells (**Figures 4A–C**).

To investigate the cause of apoptosis prone phenotype of these cells, we examined the levels of reactive oxygen species (ROS) and DNA damage in the drug resistant cells and compared to the parental cells. We found that GEM/CIS treatment resulted in accumulations of ROS in the parental, as well as in the GEM/CIS resistant CCA cell lines (**Figures 4D, E** **)**. Interestingly, the GEM/CIS resistant CCA cell lines accumulated significantly higher levels of ROS compared to the parental drug sensitive cell (**Figures 4D, E** **)**. We also detected significantly elevated level of γ-H2AX, a DNA damage marker, in the GEM/CIS resistant cell lines (**Figures 4F, G** **)**, which was associated with activation of the DDR pathway (**Supplementary Figure 2B**).

Therefore, these results indicate that the drug resistant cells adapted to survive under the persistent ROS/DNA damage-induced cellular stress and are more prone to the apoptosis imbalance. Hence, upregulations of IAPs is an essential step for the cells to evade apoptosis signal under the GEM/CIS treatment.

### Triplet LCL161 + GEM/CIS effectively prevented the emergence of GEM/CIS resistance

**Figure 5:**
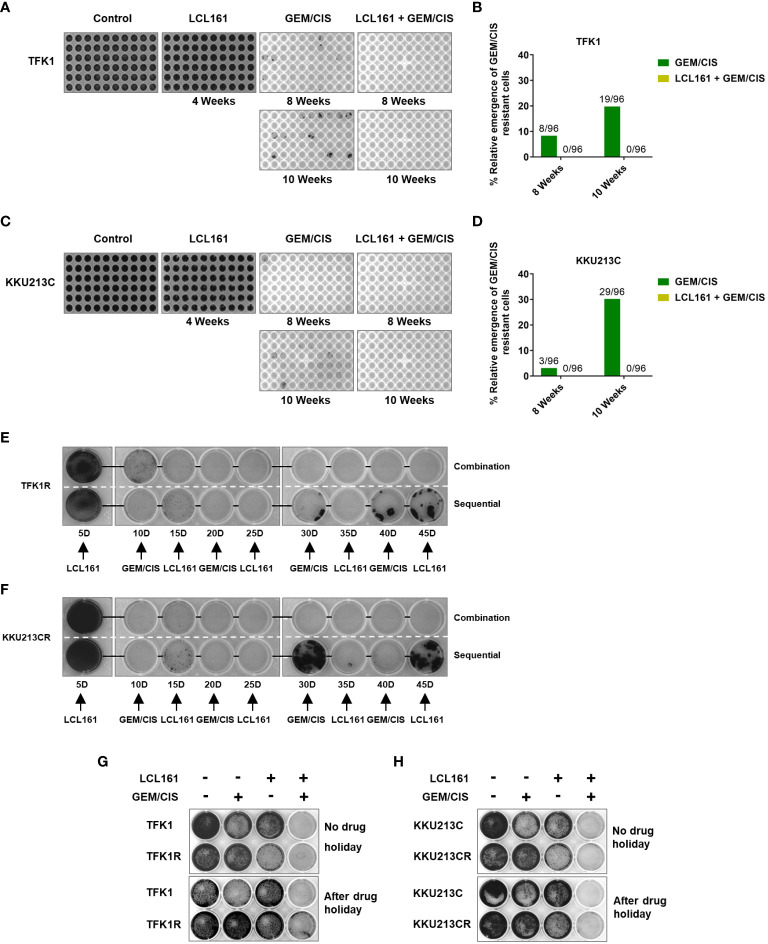
The combination of LCL161 + GEM/CIS prevents the emergence of drug-resistant cells. **(A–D)** The emergence of multidrug-resistant TFK1 and KKU213C cells under various drug treatments. TFK1 and KKU213C cells were treated with GEM/CIS, LCL161, or LCL161 + GEM/CIS for 4 weeks, 8 weeks, or 10 weeks. Emerging colonies were stained with crystal violet and imaged. The number of emerging multidrug-resistant clones in 96-well plates are shown in images **B**–**D**. (**E**, **F**) The effectiveness of triplet combination and sequential regimen is compared in TFK1R and KKU213R cells. Multidrug-resistant cells were stained with crystal violet and imaged every 5 days. (**G**, **H**) The effectiveness of the triplet therapy in TFK1, TFK1R, KKU213C, and KKU213CR cells with and without drug holiday. Cells were treated with LCL161, GEM/CIS, or LCL161 + GEM/CIS and stained with crystal violet at the indicated time points.

Since LCL161 treatment inhibited CCA cells with GEM/CIS resistance, we examined whether LCL161 can prevent the development of GEM/CIS resistance. We found that although GEM/CIS initially inhibited the growth of TFK1 and KKU213C cells, GEM/CIS-resistant cells emerged at 8 weeks, and more profoundly at 10 weeks (**Figures 5A–D**). Even though LCL161 monotherapy had no inhibitory effect on the parental TFK1 and KKU213C cells, the addition of LCL161 to GEM/CIS completely suppressed the emergence of drug-resistant cells in the long-term culture (**Figures 5A–D**).

We then compared whether LCL161 was better given sequentially or in combination with GEM/CIS (29). The results showed that LCL161 was more effective in killing drug-resistant CCA cells when given as a triplet combination compared to when given as a sequential regimen (**Figures 5E, F** **)**.

Under drug holiday, which is defined as a period during which no cancer drugs are given, we found acquired vulnerability to LCL161 in our model cell lines to be partly transient and reversed after 4 or 6 weeks of drug holiday (**Figures 5G, H** **)**. Importantly, the triplet completely suppressed all of the cells, indicating that it was effective against both transient or stable drug resistance (at 4 and 6 weeks of drug holiday – **Figures 5G, H**, respectively). These results indicate that acquired vulnerability to LCL161 can be applied to transient and stable GEM/CIS-resistant cells, and that consistent killing pressure from GEM/CIS is required to render CCA vulnerable to LCL161 treatment. These results strongly suggest the effectiveness of triplet LCL161 + GEM/CIS treatment in inhibiting and preventing an emergence of GEM/CIS-resistant CCA.

### Synergistic effect of the triplet LCL161 + GEM/CIS on drug-naïve CCA cell lines

**Figure 6:**
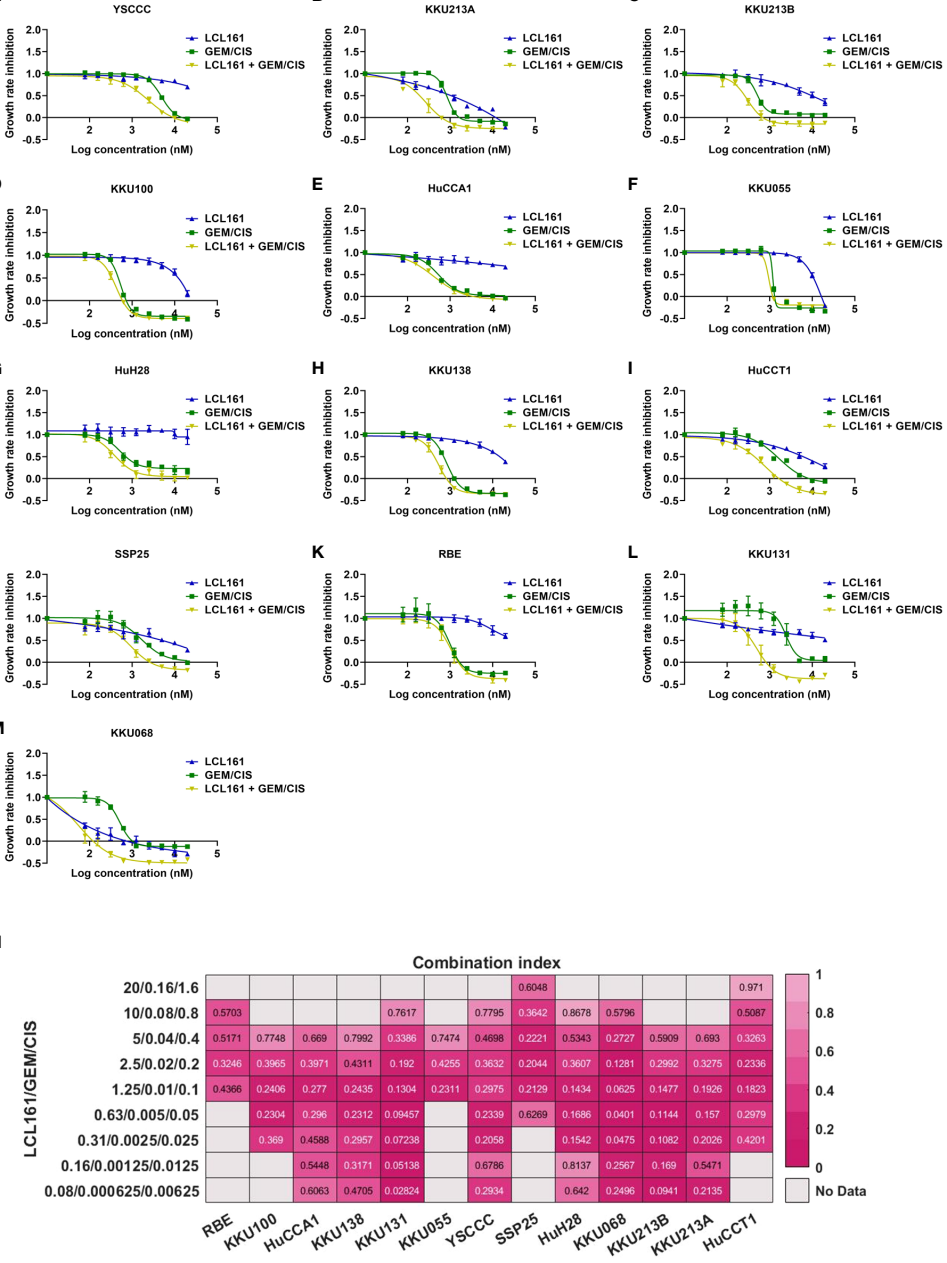
LCL161 synergistically enhances the drug efficacy of GEM/CIS in drug-naïve cell lines. **(A–M)** Dose-response curves for YSCCC, KKU213A, KKU213B, KKU100, HuCCA1, KKU055, HuH28, KKU138, HuCCT1, SSP25, RBE, KKU131, and KKU068 cell lines under LCL161, GEM/CIS, or LCL161 + GEM/CIS treatment. The triplet combination of LCL161 + GEM/CIS was more effective than LCL161 or GEM/CIS treatment. **(N)** The combination indexes (CIs) of various LCL161 + GEM/CIS combinations generated in RBE, KKU100, HuCCA1, KKU138, KKU131, KKU055, YSCCC, SSP25, HuH28, KKU068, KKU213B, KKU213A, and HuCCT1 cell lines. A CI less than 1 indicates synergism.

Since synergistic effect was observed in both GEM/CIS-resistant CCA and parental CCA counterpart cell lines, we set forth to determine whether the SMAC mimetic LCL161 synergizes with GEM/CIS in other CCA cell lines, especially in GEM/CIS-naïve CCA cells. We treated 13 CCA cell lines (YSCCC, KKU213A, KKU213B, KKU100, HuCCA1, KKU055, HuH28, KKU138, HuCCT1, SSP25, RBE, KKU131, and KKU068) with GEM/CIS, LCL161, or LCL161 + GEM/CIS for 5 days. We found that LCL161 exerted mild to no effect on most cell lines, whereas GEM/CIS was effective against most of the CCA cell lines (**Figures 6A–M**, blue and green line, respectively). Remarkably, combining LCL161 with GEM/CIS (LCL161 + GEM/CIS) potentiated GEM/CIS cytotoxicity in all CCA cells, except KKU055 and RBE, which were already hypersensitive to GEM/CIS treatment. Remarkably, combining LCL161 to GEM/CIS (LCL161 + GEM/CIS) potentiated GEM/CIS cytotoxicity in most CCA cells (except KKU055, and RBE, which are hypersensitive to GEM/CIS treatment. Addition of LCL161 mildly reduced GR50s of GEM/CIS in these 2 cell lines and the synergistic effect was observed only in some dosages), and significantly reduced the GR50s of both GEM/CIS and LCL161 (**Figures 6A–M**, **Supplementary Table 5**). In most of the CCA cell lines tested, we identified a broad range of dosages in which the triplet yielded the observed synergism (**Figure 6N**), which strongly suggests that the triplet combination yielded a synergistic effect in a broad cellular context. These results indicate that combining LCL161 with the standard GEM/CIS regimen should be expected to yield improved CCA treatment outcomes.

### LCL161 synergizes with GEM/CIS in *in vivo* drug-resistant xenograft models and patient-derived organoids

**Figure 7:**
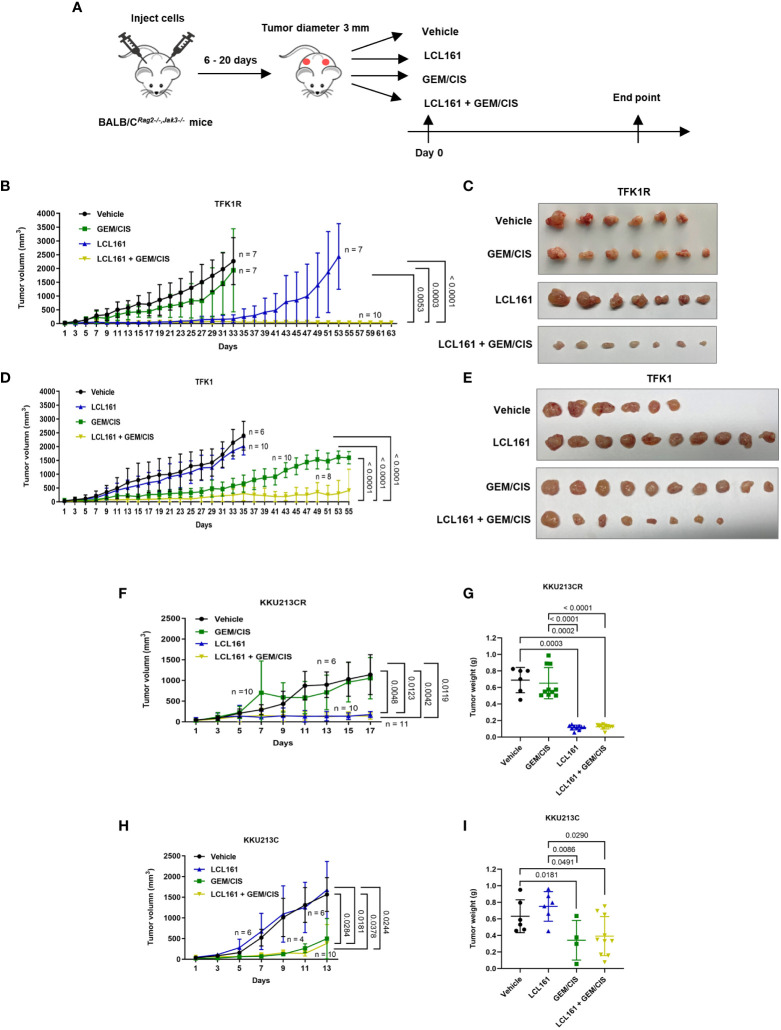
The effectiveness of triplet LCL161 + GEM/CIS therapy in *in vivo* models of GEM/CIS resistance. **(A)** BALB/C*^Rag2-/-/Jak3-/-^* mice were subcutaneously injected with GEM/CIS-resistant cells. When the tumor reached 3 mm in diameter, the mice were intraperitoneally treated with vehicle, LCL161, GEM/CIS, or triplet LCL161 + GEM/CIS. **(B)** Growth of TFK1R tumors from the beginning to the end of the experiment under various treatments. The lines show the averages of tumor size ± SD. *P*-values are shown at the right lower quadrant of the image. **(C)** Pictorial images of the tumor from the beginning to the end of treatment for each drug regimen from image **(B, D)** Growth of TFK1R tumors from the beginning to the end of the experiment under various treatments. The lines show the averages of tumor size ± SD. *P*-values are shown at the right lower quadrant of the image. **(E)** Pictorial images of the tumor from the beginning to the end of treatment for each drug regimen from image **(D, F)** Growth of KKU213CR tumors from the beginning to the endpoint, on which the untreated controls reach the critical size. The lines show the averages of tumor size ± SD. *P*-values are shown at the right lower quadrant of the image. **(G)** KKU213CR tumor weight compared among the 4 treatments. The bars indicate the average tumor weight ± SD. *P*-values are shown at the top of the 4 columns. **(H)** Growth of KKU213C tumors from the beginning to the endpoint, on which the untreated controls reach the critical size. The lines show the averages of tumor size ± SD. *P*-values are shown at the right lower quadrant of the image. **(I)** KKU213C tumor weight compared among the 4 treatments. The bars indicate the average tumor weight ± SD. *P*-values are shown at the top of the 4 columns. (n = number of tumors).

To investigate whether the triplet combination overcomes GEM/CIS resistance under the physiological condition, we generated the GEM/CIS-resistant CCA animal models by subcutaneously implanting GEM/CIS-resistant CCA cells into severely immunocompromised BALB/C*^Rag2-/-,Jak3-/-^* mice. After tumor formation was confirmed, the animals were treated with vehicle, GEM/CIS, LCL161, or the triplet (LCL161 + GEM/CIS) (**Figure 7A**). As expected, GEM/CIS had no effect on TFK1R tumor growth (**Figures 7B, C** **)**; however, LCL161 monotherapy significantly delayed the growth of the GEM/CIS-resistant TFK1R tumors. Strikingly, we found the triplet therapy to be superior to LCL161 monotherapy as demonstrated by complete suppression of the TFK1R tumor, i.e., no tumor growth was observed under the triplet therapy until the end of the experiment at 63 days.

Consistent with the *in vitro* results, growth of the parental TFK1 tumors was effectively delayed by GEM/CIS, and LCL161 monotherapy had no effect in these tumors (**Figures 7D, E** **)**. The triplet therapy effectively inhibited the growth of drug-naïve TKF1 tumors, and this effect persisted until the end of the study (day 55). Similar results were observed in another *in vivo* GEM/CIS-resistant model (KKU213CR; **Figures 7F, G** **)**, and its drug-naïve counterpart (KKU213C cells) (**Figures 7H, I** **)**. Therefore, the triplet was shown to be effective in inhibiting both GEM/CIS-resistant CCA and drug-naïve CCA cells, also that it was able to suppress the emergence of multidrug resistant cells in *in vivo* mouse models.

To extend our observation of the observed synergism of this triplet therapy in other drug-naïve CCA tumors, we tested it in two other drug-naïve xenograft models (KKU068 and KKU131). We found the triplet to be superior to GEM/CIS in inhibiting drug-naïve KKU068 and KKU131 tumors, and that it also prevented the emergence of multidrug resistant tumors (**Supplementary Figures 3A–D**). Concerning toxicity, there was no difference in weight loss between the triplet therapy and GEM/CIS therapy (**Supplementary Figures 4A, B** **)**.

**Figure 8:**
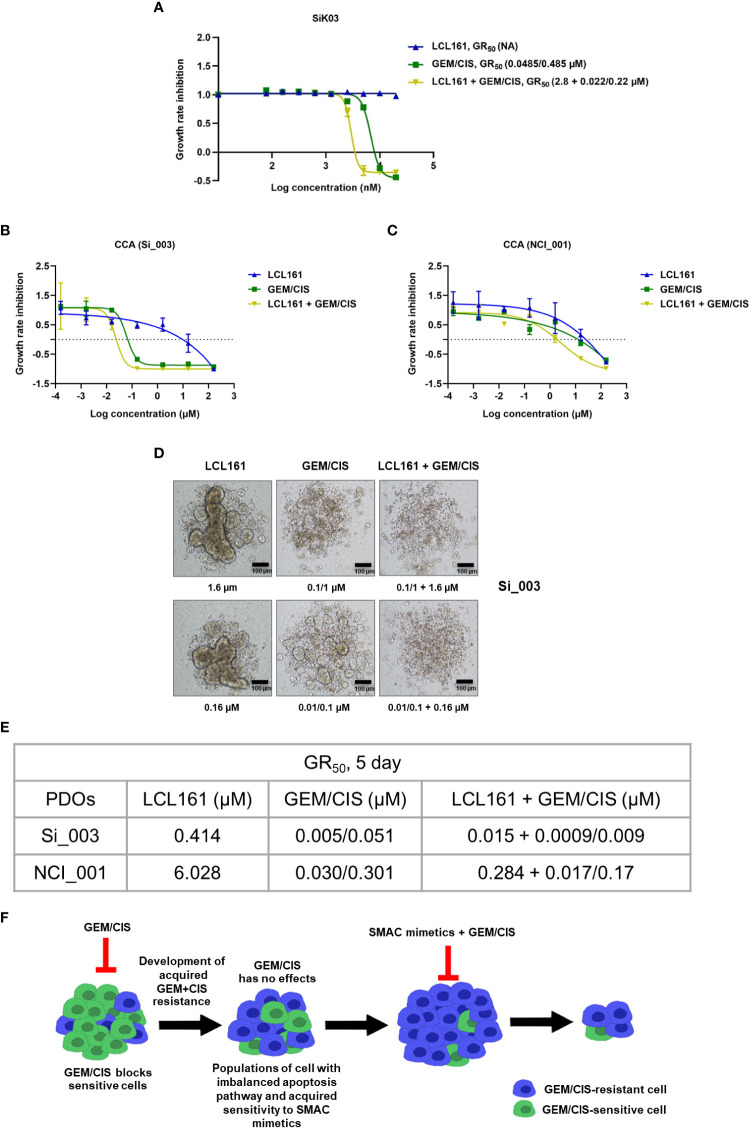
Anti-cancer effect of triplet LCL161 + GEM/CIS therapy in CCA PDOs. **(A)** Dose-response curves for LCL161, GEM/CIS, or LCL161 + GEM/CIS in a primary CCA cell line SiK03. **(B, C)** Dose-response curves for LCL161, GEM/CIS, or LCL161 + GEM/CIS in two models of PDOs (Si_003 and NCI_001). The lines represent the averages of triplicate data ± SD. The PDOs were treated for 5 days, the GR_50_ values were obtained, and **(D)** the morphologies of Si_003 PDOs treated with indicated therapies were visualized. **(E)** The 5-day GR_50_ values for LCL161, GEM/CIS, and LCL161 + GEM/CIS in PDOs. **(F)** Horizontal flow diagram describing our findings. Under GEM/CIS treatment, CCA cells adapted in an effort to survive the drug treatment by upregulating cIAP2, which resulted in a newly acquired vulnerability to SMAC mimetics, such as LCL161. Combining SMAC mimetics with the standard GEM/CIS treatment regimen was shown to promote synergistic inhibition of multidrug-resistant CCA.

Lastly, to evaluate the clinical relevance of our findings, we tested the effect of triplet therapy in a primary CCA cell line, and in 2 models of patient-derived organoids. We found the triplet to be more effective than GEM/CIS or LCL161 monotherapy in the primary CCA cell line and in both organoid models (**Figures 8A–E**). These results demonstrated the triplet therapy to be more effective in inhibiting drug-naïve CCA tumors, primary cell line, and organoids, as well as GEM/CIS-resistant tumors. In addition, the triplet was effective in preventing the emergence of multidrug resistant tumors.

## Discussion

Despite the great efforts devoted to developing new pharmacological therapies in CCA, the clinical outcomes of CCA patients remain to be poor. The resistance to anticancer treatment is a major reason for the difficulty in CCA treatment. CCA is characterized by high interpatient and intratumor heterogeneity, which makes it difficult to overcome the problem of inherited drug resistance of subclones. In addition, the acquired resistance is known to contribute to failure in the treatment of CCA in the clinic. To design new combination therapy that can reverse the mechanisms underlying multidrug resistance and effectively and ubiquitously kill or stop tumor growth, without inducing acquired resistance in CCA, seem to be an ideal therapeutic strategy.

Here our acquired vulnerability screening revealed that CCA, which acquired a resistance to GEM/CIS, developed a collateral vulnerability to the pro-apoptotic drugs, the SMAC mimetics such as LCL161 and Birinapant (**Figure 8F**). This suggests that among several possible mechanisms of chemotherapy resistance, altered cell death response may be the main resistant mechanism for the duplet GEM/CIS therapy in CCA.

Among SMAC mimetics, LCL161 and Birinapant are currently the most well-studied; their therapeutic effectiveness and the molecular mechanism of actions have been studied extensively in pre-clinical and clinical studies. LCL161 is being tested in combination with Gemcitabine + Nab-Paclitaxel in a phase I clinical trial in pancreatic cancer (NCT01934634). In a phase II study, LCL161 + paclitaxel in triple-negative breast cancer has demonstrated some promising results (30). The anti-cancer potency of Birinapant is also under investigation in several types of tumor, including colorectal cancer, hepatocellular carcinoma, glioblastoma, breast cancer (31).

From our results, addition of LCL161 to the duplet GEM/CIS can reverse the GEM/CIS resistance, and suppress emergence of multi-drug resistant CCA, *in vitro* and *in vivo.* We hypothesize that, under GEM/CIS treatment CCA cells are heavily pressured and are reliant on anti-apoptotic proteins IAPs to survive. In agreement with this hypothesis, we found that the SMAC mimetics worked best when the pressure from GEM/CIS was kept on. This explains why the triplet therapy showed the best anti-tumor result compared to GEM/CIS duplet, LCL161 monotherapy, or sequential treatment. Interestingly, we found that the GEM/CIS resistance in our cell line models may be partially transient, as shown by the reversal of the phenotypes from LCL161-sensitive to LCL161-resistance after drug holiday. Nevertheless, the triplet was still able to eliminate the cells with reversed phenotype, indicating that the triplet is effective against both reversible and non-reversible cell re-profiling. This is in keeping with the results that the triplet therapy was effective in many drug-naïve cell lines, and PDOs that we tested.

Further work is needed to explain why the triplet was very effective in drug-naïve CCA models, in which LCL161 monotherapy had mild or no effect. One possible explanation is that in most of the CCA cells, a temporal expression of IAP proteins may be needed under GEM/CIS therapy for the CCA to escape cell death (32). SMAC mimetics may interfere with that transient expression, therefore, prohibiting the survival of the CCA cells.

The treatment of many tumors involves drug combinations. Such combinations are commonly chosen primarily due to their non-overlapping mechanism of action or toxicity. These approaches may need to be revised, since virtually patients only benefit from independent drug action, without drug synergy or additivity (33). We argue here that the candidate selection based on acquired vulnerability screening may be an attractive alternative approach. All in all, from our findings, we proposed that the triplet LCL161 + GEM/CIS therapy may be attractive as a first-line therapy for cholangiocarcinoma.

## Data availability statement

The original contributions presented in the study are included in the article/**Supplementary Material**. Further inquiries can be directed to the corresponding author.

## Ethics statement

The animal study protocol #009/2564 was reviewed and approved by Faculty of Medicine Siriraj Hospital, Mahidol University – Institute Animal Care and Use Committee.

## Author contributions

SP, OS, BP, CJ, JC, AC, PM-K, KMK, and MS performed the experiments. KK, SO, SS, and SJ supervised and provided critical comments, the experimental framework, and the experimental design. RC, and SO provided important resources and clinical specimens. SP, CJ, JC, AC, and SJ analyzed and interpreted the data. SP and SJ wrote and edited the manuscript. SJ obtained financial support for the study. All authors contributed to the article and approved the submitted version.
