# Corrigendum: Combining the SMAC mimetic LCL161 with Gemcitabine plus Cisplatin therapy inhibits and prevents the emergence of multidrug resistance in cholangiocarcinoma

**DOI:** 10.3389/fonc.2022.1124912

**Published:** 2023-01-06

**Authors:** Sunisa Prasopporn, Orawan Suppramote, Ben Ponvilawan, Chanette Jamyuang, Jantappapa Chanthercrob, Amphun Chaiboonchoe, Pimkanya More-Krong, Kamonchanok Kongsri, Monthira Suntiparpluacha, Rawisak Chanwat, Krittiya Korphaisarn, Seiji Okada, Somponnat Sampattavanich, Siwanon Jirawatnotai

**Affiliations:** ^1^ Siriraj Center of Research Excellence for Precision Medicine and Systems Pharmacology, Faculty of Medicine Siriraj Hospital, Mahidol University, Bangkok, Thailand; ^2^ Department of Pharmacology, Faculty of Medicine Siriraj Hospital, Mahidol University, Bangkok, Thailand; ^3^ Princess Srisavangavadhana College of Medicine, Chulabhorn Royal Academy, Bangkok, Thailand; ^4^ HepatoPancreato-Biliary Surgery Unit, Department of Surgical Oncology, National Cancer Institute, Bangkok, Thailand; ^5^ Division of Medical Oncology, Department of Medicine, Faculty of Medicine Siriraj Hospital, Mahidol University, Bangkok, Thailand; ^6^ Division of Hematopoiesis, Joint Research Center for Human Retrovirus Infection, Kumamoto University, Kumamoto, Japan

**Keywords:** SMAC mimetics, LCL161, Gemcitabine plus Cisplatin therapy, multidrug resistance, cholangiocarcinoma, acquired vulnerability

In the published article, there was an error in the Funding statement. There was one missing funding agency from our manuscript. The correct Funding statement appears below.

