# Supplementary material for: Combining the SMAC mimetic LCL161 with Gemcitabine plus Cisplatin therapy inhibits and prevents the emergence of multidrug resistance in cholangiocarcinoma
Source: Front Oncol. 2022 Nov 30;12:1021632. doi: 10.3389/fonc.2022.1021632 (PMC9748615; doi:10.3389/fonc.2022.1021632)
Supplement: Supplementary file 1 [file DataSheet_1.pdf]

A

GR$_{50}$, 5 day	
CCA cell lines	GEM/CIS (μM)
TFK1	0.00317/0.03309
TFK1R	0.0321/0.298
KKU213C	0.010126/0.10126
KKU213CR	0.03402/0.3402
KKU068	0.0014/0.0162
KKU068R	0.0168/0.16813
SSP25	0.011533/0.116018
SSP25R	0.0448/0.4484

B

GR$_{50}$, 5 day		
CCA cell lines	GEM (μM)	CIS (μM)
KKU068	0.009	3.38
KKU213A	0.02	3.29
KKU213B	0.018	3.48
KKU131	0.03	3.64
KKU138	0.0053	0.383
SSP25	0.011	0.968
RBE	0.006	1.3
KKU055	0.027	0.402
KKU213C	0.024	2.98
TFK1	0.016	1.47

Supplementary Table 1 (A) five-day GR$_{50}$ in parental cells (TFK1, KKU213C, KKU068, and SSP25 cells) and resistant cells (TFK1R, KKU213CR, KKU068R, and SSP25R cells) from GEM/CIS dose-response curves. (**B**) five-day GR$_{50s}$ of GEM and CIS monotherapy in tested CCA cell lines.

Supplementary Figure 1

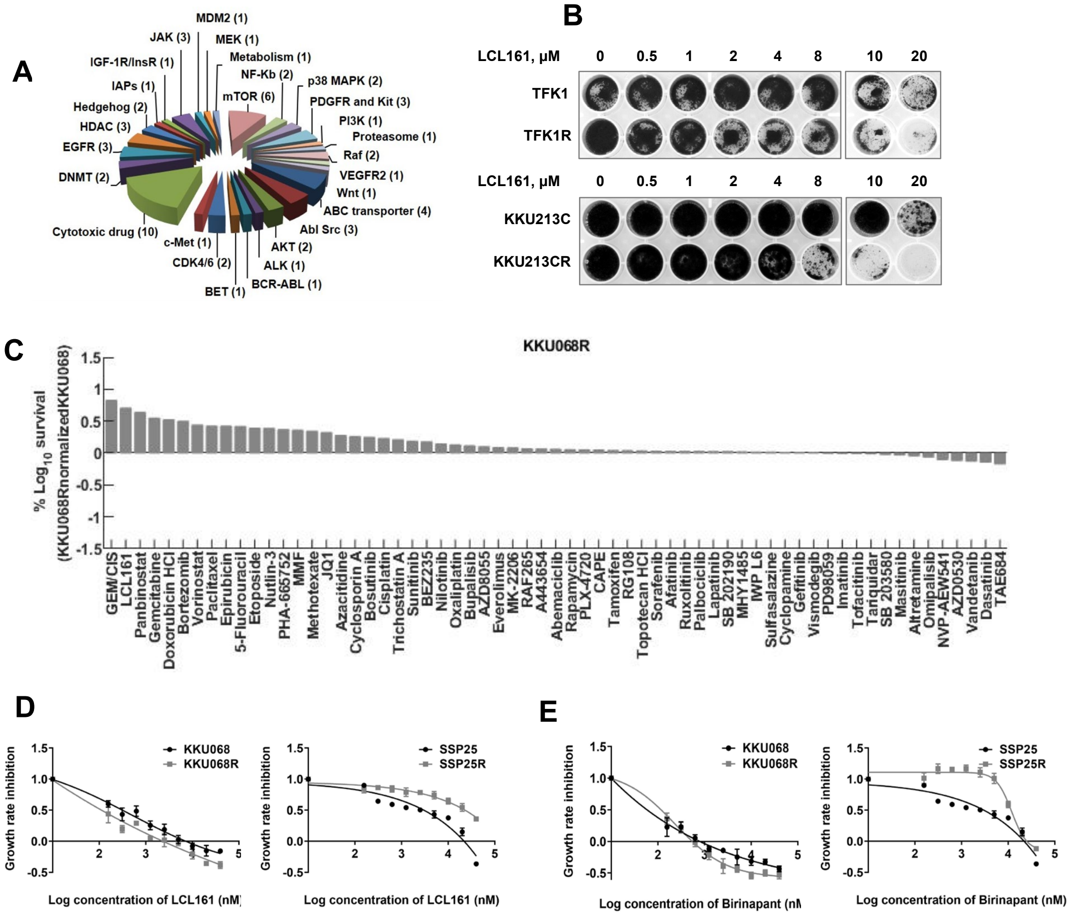

Supplementary Figure 1. (**A**) Pie chart indicating the classes of drugs used in the acquired vulnerability screenings. The library consists of 62 cancer drugs that cover 28 specific cancer driver proteins. (**B**) Colony survival assays of LCL161 – TFK1 *vs.* TFK1R cells, and KKU213C *vs.* KKU213CR cells. (**C**) The result of acquired vulnerability screening in KKU068R cells. (**D**) LCL161 dose-response curves for KKU068 and KKU068R, and SSP25 and SSP25R cells. (**E**) Birinapant dose-response curves for KKU068 and KKU068R, and SSP25, and SSP25R cells.

GR_{50}, 5 day		
CCA cell lines	LCL161 (μM)	Birinapant (μM)
TFK1	11.947	16.958
TFK1R	0.232	0.099
KKU213C	15.300	12.473
KKU213CR	3.546	6.376
KKU068	0.317	0.035
KKU068R	0.0511	0.111
SSP25	2.767	2.767
SSP25R	23.036	11.737

Supplementary Table 2. Five-day GR_{50} in parental cells (TFK1, KKU213C, KKU068, and SSP25 cells) and pooled resistant cells (TFK1R, KKU213CR, KKU068R, and SSP25R cells) from LCL161 and birinapant dose-response curves.

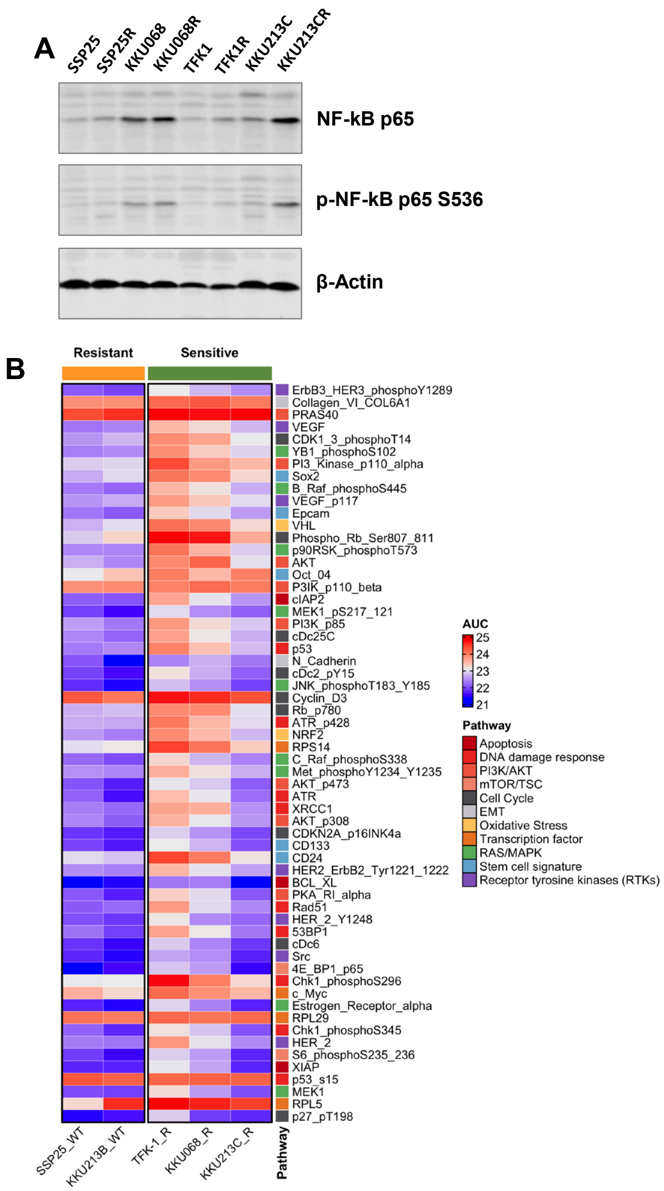

Supplementary Figure 2. (**A**) Western blot analysis of NF-kB and p-NF-kB S536 in parental and pooled resistant cell lines. β-actin was used as a protein loading control. (**B**) A heatmap representation of differential expressions of proteins in LCL161-sensitive (green) and resistant (yellow) cell lines. AUC values of the indicated protein (to the right of the panel) at time point 48 and 72 hrs after GEM/CIS treatment), generated from RPPA, are shown-red indicates higher expression; blue indicates lower expression. Row and column of this heatmap plot are top 60 antibodies (from 103 antibodies) with ranked VIP scores of 5 cell lines. Each protein is annotated by its pathway.

GR_{50}, 3 day	
CCA cell lines (KD)	GEM/CIS (μM)
TFK1	0.0093/0.0934
TFK1R_siRNA Control	0.0948/0.9476
TFK1R_sicIAP2#1	0.0407/0.4071
TFK1R_sicIAP2#2	0.02448/0.248
TFK1R_sicIAP2#3	0.0444/0.4441

Supplementary Table 3. Three-day GR_{50} in cIAP2 knockdown and control TFK1R cells from GEM/CIS dose-response curves.

GR_{50}, 3 day	
CCA cell lines (KD)	GEM/CIS (µM)
KKU213C	0.0051/0.0508
KKU213C_siRNA Control	0.0756/0.7556
KKU213C_sicIAP2#1	0.0442/0.4424
KKU213C_sicIAP2#2	0.0342/0.342
KKU213C_sicIAP2#3	0.044/0.4401

Supplementary Table 4. Three-day GR_{50} in cIAP2 knockdown and control KKU213CR cells from GEM/CIS dose-response curves.

GR_{50}, 5 day			
CCA cell lines	LCL161 (μM)	GEM/CIS (μM)	LCL161 + GEM/CIS (μM)
KKU068	0.02	0.004/0.038	0.037 + 0.0003/0.003
KKU213A	1.079	0.006/0.066	0.185 + 0.001/0.015
KKU213B	9.53	0.004/0.041	0.253 + 0.002/0.02
KKU100	11.11	0.0038/0.038	0.33 + 0.0026/0.027
HuCCA1	NA	0.004/0.042	0.36 + 0.0028/0.029
KKU131	35.33	0.022/0.223	0.37 + 0.003/0.03
HuH28	NA	0.005/0.056	0.41 + 0.003/0.03
KKU138	14.58	0.005/0.057	0.41 + 0.0033/0.03
HuCCT1	6.29	0.012/0.121	0.41 + 0.0033/0.03
SSP25	5.12	0.014/0.141	0.61 + 0.005/0.048
RBE	NA	0.007/0.075	0.807 + 0.006/0.065
KKU055	9.75	0.008/0.082	0.86 + 0.007/0.069
YSCCC	NA	0.037/0.378	1.74 + 0.014/0.139

Supplementary Table 5. Five-day GR_{50} in KKU068, KKU213A, KKU213B, KKU100, HuCCA1, KKU131, HuH28, KKU138, HuCCT1, SSP25, RBE, KKU055, and YSCCC cells from LCL161, GEM/CIS, or LCL161 + GEM/CIS dose-response curves.

Supplementary Figure 3

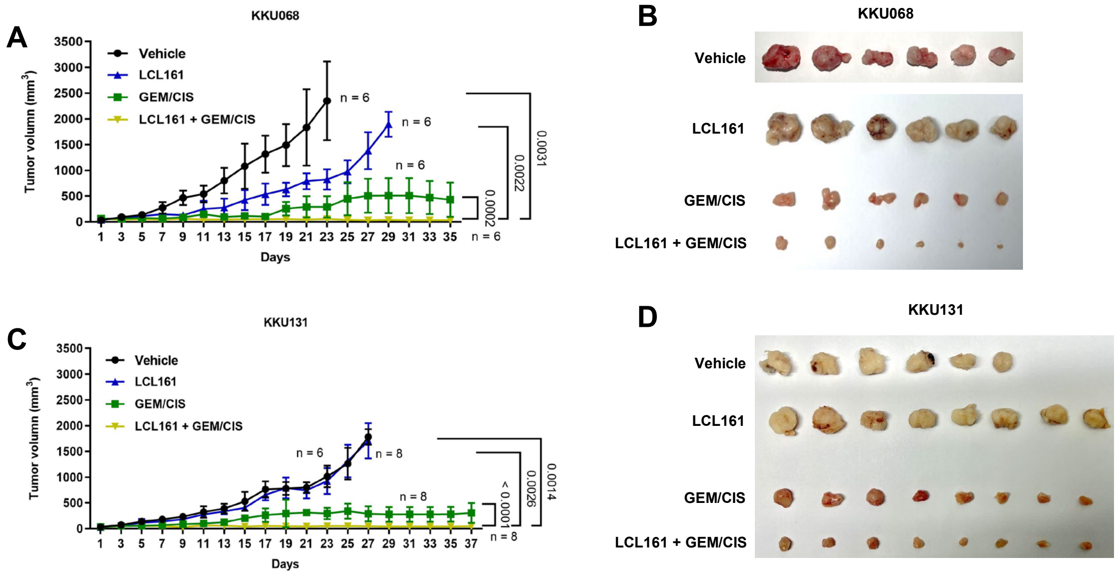

Supplementary Figure 3. The effectiveness of triplet LCL161 + GEM/CIS treatment in *in vivo* drug-naïve models. (**A**) The growth of KKU068 tumors from the beginning to the end of the experiment under various treatments. The lines show the averages of tumor size ± SD. *P*-values are shown at the right lower quadrant of the image. (**B**) Pictorial images of the tumor from the beginning to the end of treatment for each drug regimen from image **A**. (**C**) Growth of KKU131 tumors from the beginning to the end of the experiment under various treatments. The lines show the averages of tumor size ± SD. *P*-values are shown at the right lower quadrant of the image. (**D**) Pictorial images of the tumor from the beginning to the end of treatment for each drug regimen from image **C**.

Supplementary Figure 4

A

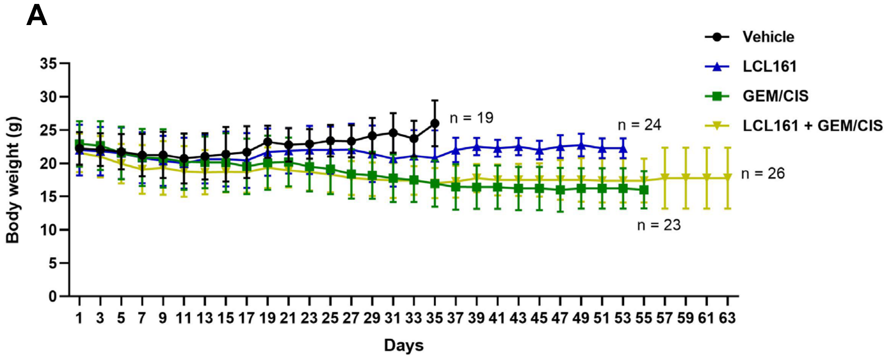

B

Groups	% Weight loss (average)
Vehicle	0
LCL161	0
GEM/CIS	17.6
LCL161 + GEM/CIS	16.13

Supplementary Figure 4. (**A**) Average body weight of mice from the beginning to the end of the experiment in each treatment group (n = mice). (**B**) Average percentage weight loss of mice in each treatment group.
